# Synthetic bovine NK-lysin-derived peptide (bNK2A) does not require intra-chain disulfide bonds for bactericidal activity

**DOI:** 10.1371/journal.pone.0218507

**Published:** 2019-06-19

**Authors:** Rohana P. Dassanayake, Shollie M. Falkenberg, Eric M. Nicholson, Robert E. Briggs, Fred M. Tatum, Vijay K. Sharma, Timothy A. Reinhardt

**Affiliations:** 1 Ruminant Diseases and Immunology Research Unit, National Animal Disease Center, Agricultural Research Service, United Sates Department of Agriculture, Ames, IA, United States of America; 2 Virus and Prion Research Unit, National Animal Disease Center, Agricultural Research Service, United States Department of Agriculture, Ames, IA, United States of America; 3 Food Safety and Enteric Pathogens Research Unit, National Animal Disease Center, ARS-USDA, Ames, IA, United States of America; Fondazione Pisana per la Scienza, ITALY

## Abstract

Bovine NK-lysins are cationic antimicrobial proteins found predominantly in the cytosolic granules of T lymphocytes and NK-cells. NK-lysin-derived peptides show antimicrobial activity against both Gram positive and Gram negative bacteria. Mature NK-lysin protein has six well-conserved cysteine residues. This study was performed to assess whether synthetic bovine NK-lysin-derived peptide (bNK2A) forms disulfide bonds and whether disulfide bonds were essential for bNK2A antimicrobial activity. Two 30-mer bNK2A peptides were synthesized: one with two original cysteines and an analog with cysteines substituted with two serines. Mass spectrometry revealed lack of disulfide bonds in original peptide while CD spectrophotometry showed both peptides have similar α-helical structures. Since both peptides were equally inhibitory to *Histophilus somni*, disulfide bonds appeared dispensable for synthetic bNK2A peptide antibacterial activity.

## Introduction

Antimicrobial peptides (AMPs) are evolutionary conserved molecules that are part of the innate defense mechanism and are produced by virtually all living organisms [1, 2]. Bovine NK-lysins have been identified as cationic antimicrobial proteins and are mainly produced by T lymphocytes and natural killer (NK) cells [3]. Bovine NK-lysin is structurally and functionally similar to porcine NK-lysin and human granulysin [3–5]. Although pigs and humans have only a single functional NK-lysin or granulysin gene, cattle have four functional NK-lysin genes (*NK1*, *NK2A*, *NK2B*, and *NK2C*) [6]. Both NK-lysins and granulysin show structural similarities to saposin-like protein (SAPLIP) family of lipid-binding proteins [5, 7, 8]. The sap domain of SAPLIPs is comprised of four to five α-helices connected by loops and six well-conserved cysteine residues which form three intra-chain disulfide bonds [9]. These disulfide bonds appear to stabilize the tertiary structure and also the biological function of SAPLIPs [9].

Conflicting reports have emerged on the importance of intra-chain disulfide bonds for effective antimicrobial activity of human granulysin, porcine NK-lysin, hemipteran insect (*Podisus maculiventris*) thanatin, and protozoan parasite (*Entamoeba histolytica*) NK-lysin [10–13]. However, no such information is currently available on bovine NK-lysins, which is important for our ongoing efforts to explore bovine NK-lysin-derived peptides as therapeutic agents to control bovine respiratory disease complex (BRDC) in cattle. Therefore, the objectives of this study were to assess whether synthetic bovine NK-lysin-derived peptide forms intra-chain disulfide bonds and whether such disulfide bonds were essential for NK-lysins antimicrobial activity.

## Materials and methods

### Peptide synthesis

A 30-mer synthetic peptide corresponding to the functional region helices 2 and 3 of bovine NK-lysin NK2A with the two original cysteine residues at number 10 and 20 positions (bNK2A-C10C20: TVIEVASKMCSKMRLLKGLCKSITKRFLRR) was synthesized (Peptide 2.0 Inc, Chantilly, VA) and supplied as trifluoroacetate salt with over 95% purity [14, 15]. An analog peptide with the two cysteine residues replaced with two serine residues (bNK2A-S10S20: TVIEVASKMSSKMRLLKGLSKSITKRFLRR) was also synthesized. Both peptides were dissolved in Dulbecco’s Phosphate Buffered Saline (PBS, pH 7.4) and stored at -20°C until used. The concentrations of both peptides were determined by quantitative amino acid analysis at the Protein Chemistry Laboratory, Texas A&M University.

### Circular dichroism assay

Circular dichroism (CD) assay of bNK2A-C10C20 and bNK2A-S10S20 peptides was performed as described previously [15, 16]. Briefly, both peptides (20 μM final concentrations) were diluted in 50 mM sodium phosphate buffer (NaPB, pH 7.4) with 50% trifluoroethanol (TFE) or without TFE and placed in a 1 mm path-length quartz cuvette (final volume = 300 μL). The α-helical structures of both peptides were then evaluated using a Jasco J-815 CD spectrophotometer (Jasco, Easton, MA). Measurements were taken every 0.1 nm from 250 to 200 nm with six accumulations at room temperature using automated baseline correction. Raw data in millidegrees was converted to molar ellipticity.

### Mass spectrometry analysis

Approximately 7.0 μg of original bNK2A-C10C20 peptide was aliquoted into four tubes (10 μL each) and 20 mM triethylammonium borocarbonate buffer, pH 8.0 (13 μL each) was added to each tube. Two microliters of tris(2-carboxyethyl)phosphine (TCEP; final concentration = 50 mM) were added to two tubes and PBS to the other two tubes and samples were incubated at room temperature for 10 min. An alkylating agent iodoacetamide (IAA) was added to one of the PBS and TCEP tubes (IAA; 110 mM final concentration) and incubated at room temperature for additional 60 min. Samples were diluted in PBS and matrix-assisted laser desorption/ionization (MALDI-TOP) mass spectrometry analysis was performed using Q Exactive Hybrid Quadrupole-Orbitrap Mass Spectrometer (ThermoFisher Scientific) at the Protein Facility, Iowa State University.

### Antimicrobial killing assay

Bovine pneumonic *Histophilus somni* isolates 91 and 2336 were grown in trypticase soy agar plates supplemented with 5% defibrinated sheep blood (TSA II, Becton, and Dickinson Co., Sparks, MD) at 37°C in a humidified atmosphere of 7.5% CO_2_. Antimicrobial killing assay was performed as described by us previously [14]. Briefly, *H*. *somni* isolates grown on TSA sheep blood agar plates for approximately 16 hrs were collected using a cotton swab and diluted in Columbia broth to an optical density at 600 nm of 0.8 (~1 × 10^9^ colony forming units per milliliter (CFUs/mL)). The bacterial suspension was further diluted in PBS (1:200) to obtain ~5 × 10^6^ CFUs/mL. One hundred microliter aliquots of bacterial suspension (~5 × 10^5^ CFUs) were placed in a non-tissue culture treated flat-bottom 96-well plate and gently mixed with 20 μL of diluted bNK2A-C10C20 and bNK2A-S10S20 peptides (1, 2, 5 and 20 μM final concentrations) or PBS (negative control). The plate was covered with a lid and incubated at 37°C in a humidified atmosphere of 7.5% CO_2_ with constant shaking at 50 rpm for 60 min. Bacterial samples were serially diluted (10 fold) in PBS and 100 μL aliquot of each dilution was spread on TSA sheep blood agar plates in triplicate and incubated at 37°C for 2 days. Bacterial colonies were enumerated for each dilution. The percentage of remaining viable bacteria in bNK2A peptides treated samples was calculated by dividing the average number of CFUs/mL in each sample by the average number of CFUs/mL in the corresponding negative control × 100. Antimicrobial killing assays were repeated at least three times.

### Treatment of bNK2A-C10C20 with DTT

Twenty micro molar final concentration of original bNK2A-C10C20 peptide was pre-incubated with 0.2, 0.5, and 1.0 mM Dithiothreitol (DTT) at 37°C for 20 min [10, 11]. Peptide-DTT mixture was then added to 100 μL of *H*. *somni* (~5 × 10^5^ CFUs) and further incubated at 37°C in a humidified atmosphere of 7.5% CO_2_ for 60 min and CFUs assay was performed as described for antimicrobial killing assay.

### Two-color flow cytometry

To distinguish dead bacteria from live bacteria following incubation with bNK2A peptides, *H*. *somni* was stained using LIVE/DEAD *Bac*Light bacterial viability kit (Cat no. L13152; ThermoFisher Scientific, Carlsbad, CA) as described by us previously [14]. Briefly, ~1 × 10^8^ CFUs of *H*. *somni* suspension in 100 μL PBS was placed in a flat-bottom 96-well plate and 20 μM bNK2A peptides were added and incubated at 37°C in a humidified atmosphere of 7.5% CO_2_ with constant shaking for 30 min. Untreated and heat-killed (56°C for 30 min) *H*. *somni* were used as live and dead controls, respectively. Fifty microliters of Syto 9 and 50 μL of propidium iodide (approximate final concentrations of Syto 9 and propidium iodide (PI) were 6 μM and 30 μM, respectively) were added to each well and incubated for additional 15 min. Two-color flow cytometric analyses was performed using a BD LSRII flow cytometer (BD Biosciences). Both Syto 9 and PI were excited at 488 nm laser beam and the emission signals were detected using a 530/30 nm and 575/25 nm long-pass filters, respectively. At least 10,000 events were collected for data analysis and relative live/dead bacterial changes were determined using FlowJo software (FlowJo LLC, Ashland, OR). Means and standard deviations of percentages of dead *H*. *somni* were calculated from three experiments.

### Hemolytic assay

The hemolytic activity of bNK2A peptides was determined using bovine red blood cells (RBCs) as described previously [15, 17]. Briefly, 10 mL of cattle blood samples were collected into acid citrate dextrose anticoagulant, transferred to 15 mL tubes and centrifuged (1000 × *g* for 10 min) to remove plasma and buffy coat. RBC pellets were washed twice with PBS and resuspended in PBS to obtain 2.5% hematocrit. One hundred microliters of RBC suspensions were placed into a flat-bottom 96-well plate and mixed gently with 20 μL of bNK2A peptides in triplicate (5 and 20 μM final concentration, L_Exp_). The plate was kept in incubator with 5% CO_2_ at 37°C in a humidified atmosphere and incubated for 60 min. The plate was centrifuged and 100 μL of each supernatant was transferred to a new flat-bottom 96-well plate and absorbance was measured at 405 nm wavelength using FlexStation 3 Multi-Mode Microplate Reader (Molecular Devices, San Jose, CA). Triton X-100 (0.1% (v/v) final concentration, (L_Tx100_) -treated RBCs were used as a positive control (100% lysis) and PBS-treated RBCs (L_0_) were used as a negative control. The percentage of hemolysis was calculated using the formula (L_Exp_-L_0_)/L_Tx100_-L_0_) × 100. Means and standard deviations were calculated from two independent experiments.

### Statistical analysis

Student’s *t*-test was used to compare mean percentage of remaining viable *H*. *somni* in control and bNK2A peptides treated samples. The term significant indicates *P* value of less than 0.05.

## Results and discussion

**Fig 1:**
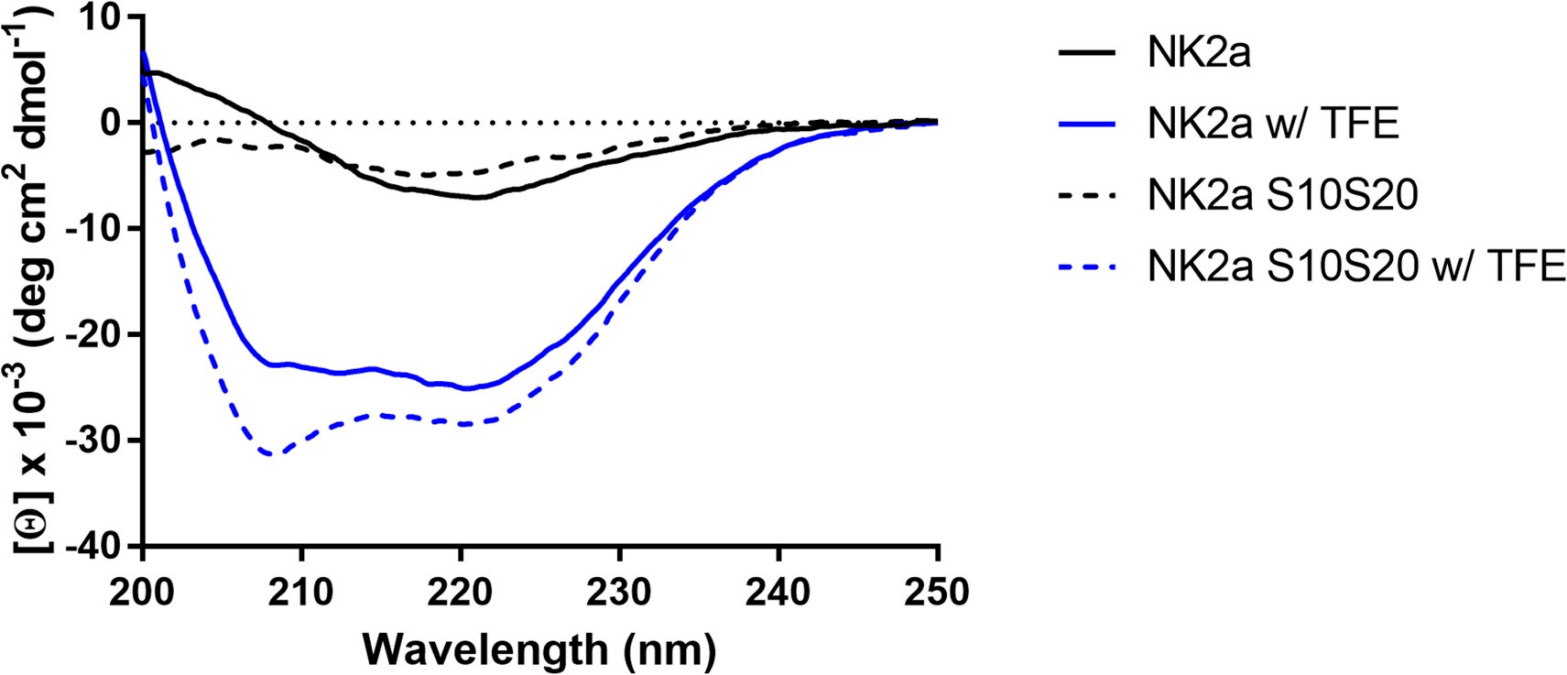
The original bNK2A-C10C20 and an analog bNK2A-S10S20 peptides showed similar α-helical confirmations. Circular dichroism (CD) spectral analysis of the peptides (20 μM final concentrations) was performed at room temperature using Jasco J-850 CD spectrophotometer in the presence of or absence of 50% TFE. Measurements were taken every 0.1 nm from 200 nm to 250 nm. Shown graphs are the means of six accumulations after baseline correction for each peptide.

The potent antimicrobial activity of synthetic bovine NK-lysin-derived peptides such as NK1, NK2A, NK2B and NK2C on bovine pathogenic *H*. *somni* pneumonic isolates have been previously reported by us [14]. Since all four synthetic 30-mer bovine NK-lysin peptides have conserved two cysteines at position 10 (helix 2) and 20 (helix 3) and NK2A showed the strongest bactericidal activity not only against *H*. *somni* [14] but also against other BRDC pathogens [15, 18], NK2A peptide was selected for the present study. Intra-chain disulfide bonds are important for the structural stability of the α-helices in the Sap domains of SAPLIPs [9, 12]; therefore we first determined the α-helical structures of both peptides by CD assay using a Jasco J-815 CD spectrophotometer. Both peptides exhibited similar and characteristic CD spectral changes indicative of α-helical structures with TFE (maxima near 200 nm with double minima near 208 nm and 222 nm) (Fig 1). Both peptides, diluted in NaPB, showed random coil structures (Fig 1). These findings suggested that intra-chain disulfide bond formation was not essential to form or stabilize α-helix confirmation of both synthetic bNK2A peptides.

**Fig 2:**
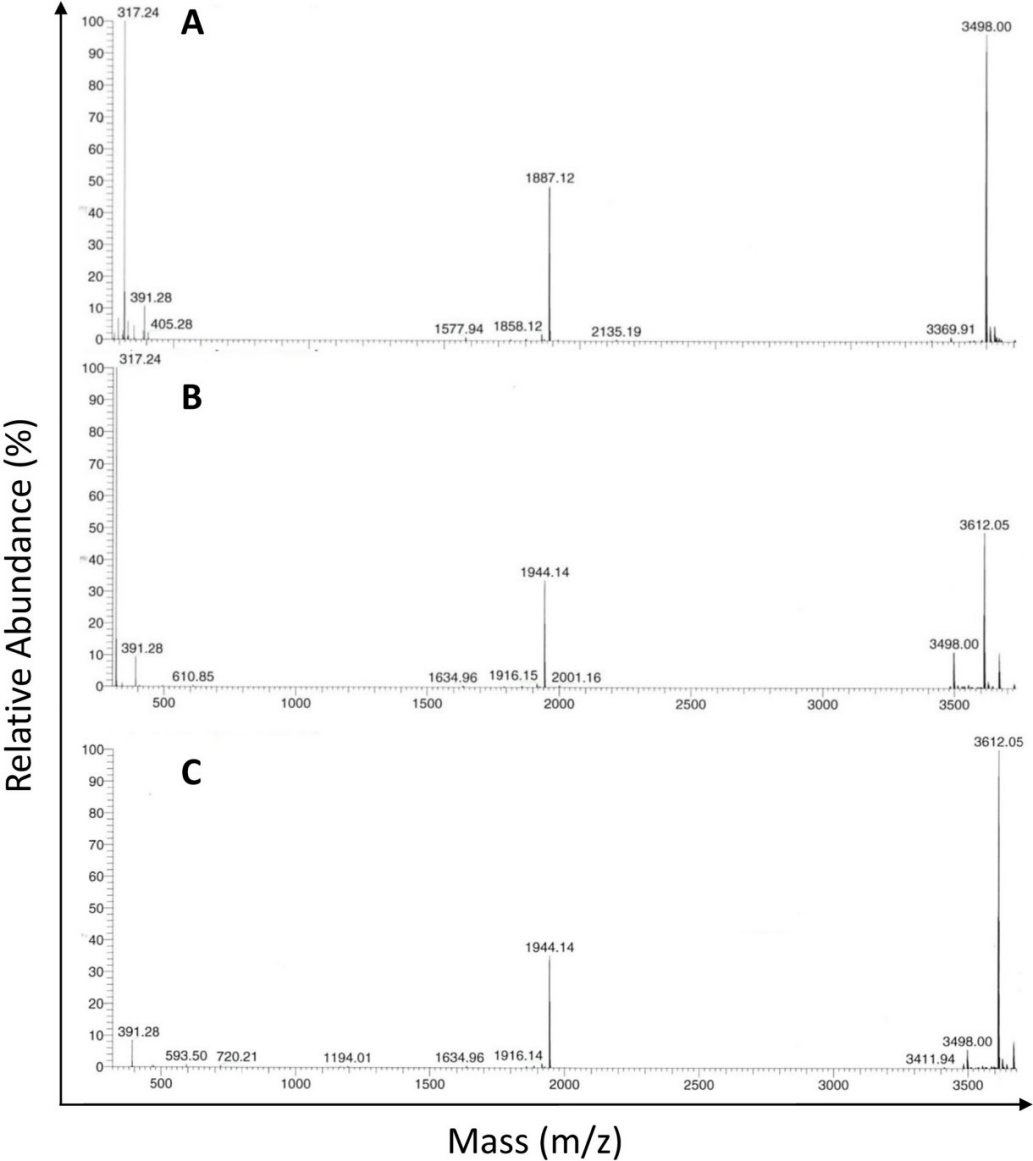
MALDI-TOP mass spectrum analysis revealed the lack of disulfide bond formation in bNK2A-C10C20 peptide. The original bNK2A-C10C20 peptide incubated with PBS (A), PBS followed by IAA (B), or TCEP followed by IAA at room temperature for 60 min and molecular masses of the peptide in each sample were analyzed using Q Exactive Hybrid Quadrupole-Orbitrap Mass Spectrometer.

Because the CD spectroscopy assay does not provide any information on disulfide bonding, an assay to determine if the original peptide forms intra-chain disulfide bond was conducted with the original bNK2A peptide. The expected molecular mass of bNK2A-C10C20 peptide without intra-chain disulfide bond is 3497.42 Da. The peptide incubated only with disulfide bond reducing agent TCEP or PBS showed a mass of 3498.00 Da suggesting the lack of intra-chain disulfide bond (Fig 2A). An alkylating agent iodoacetamide (IAA) is known to covalently bind with reduced thiol groups. If amide group (57 Da) of IAA had bound to each of the two cysteines in bNK2A-C10C20, the mass of the peptide is expected to increase by 114 Da. Indeed, the peptide incubated with PBS or TCEP followed by IAA increased in its molecular mass by 114 Da to 3612.05 Da (Fig 2B and Fig 2C). These findings strongly suggested the lack of intra-chain disulfide bond formation in the synthetic bNK2A-C10C20 peptide.

**Fig 3:**
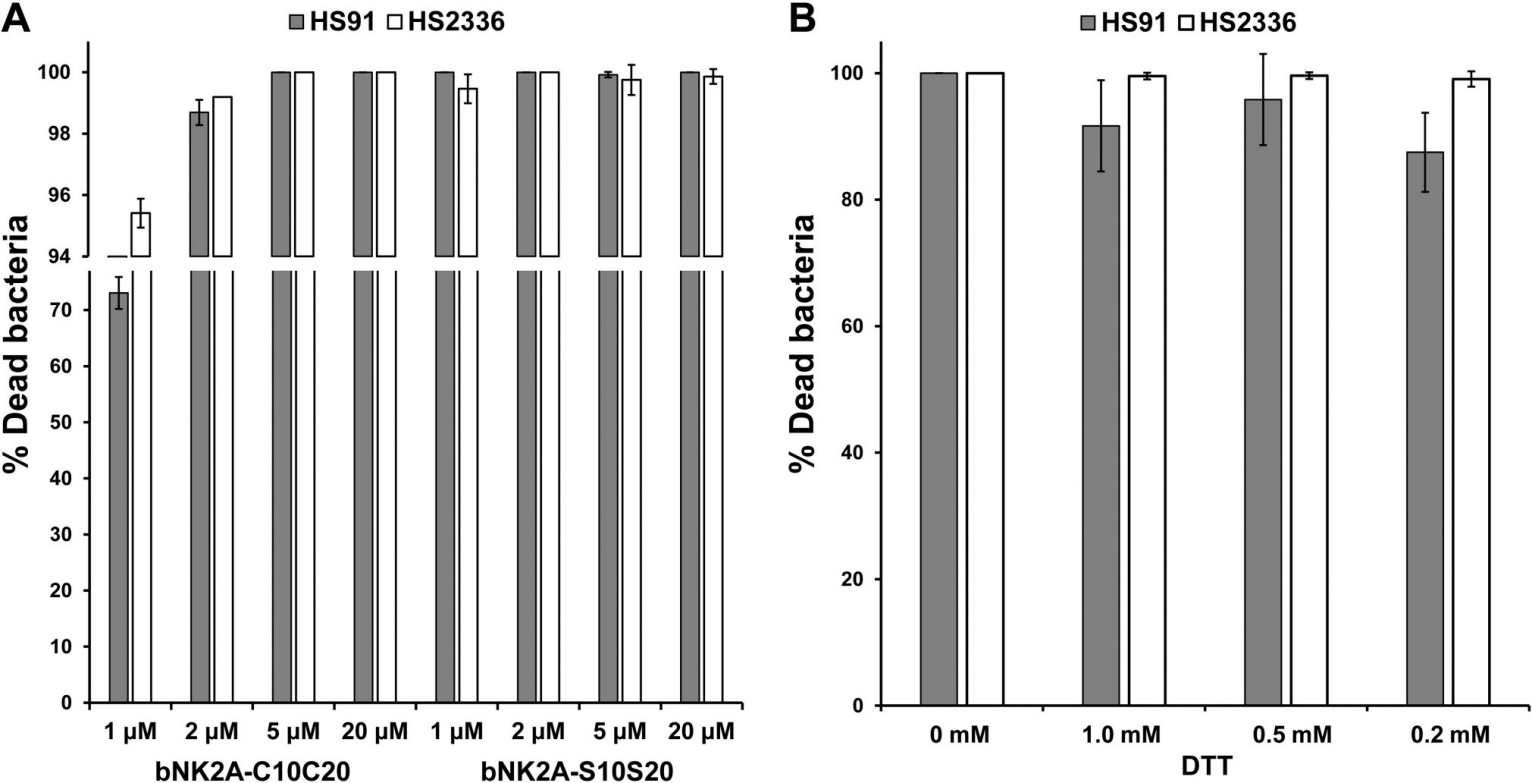
Both original bNK2A-C10C20 and an analog bNK2A-S10S20 peptides displayed strong antimicrobial activities against *Histophilus somni*. (A). Two bovine *H*. *somni* pneumonic isolates (91 and 2336) were incubated with indicated concentrations of peptides or PBS (control) and incubated at 37°C in a humidified atmosphere of 7.5% CO_2_ for 60 min. (B). The original bNK2A-C10C20 peptide (20 μM) was pre-incubated with 0.2, 0.5, and 1.0 mM DTT for 20 min before incubation with *H*. *somni* (91 and 2336). Results shown are the means and SD of three independent experiments.

Although the lack of intra-chain disulfide bonds in original bNK2A-C10C20 peptide was now confirmed, it was also unknown whether the original two cysteines are needed for bNK2A effective antimicrobial activity. Therefore, antimicrobial activity assay was performed with both peptides against two *Histophilus somni* isolates (91 and 2336) [14]. We did not find any significant difference in bactericidal activity between two peptides at 2, 5 and 20 μM final concentrations (*P* >0.01) and both peptides showed ≥98% killing activity against both isolates of *H*. *somni* (Fig 3A). However, significant difference in bacterial killing activity between two peptides was observed at 1 μM final concentration (*P*<0.001). Strong bactericidal activity was also observed when original bNK2A-C10C20 peptide was pre-incubated for 20 min with 0.2, 0.5, and 1.0 mM Dithiothreitol (DTT), which is an effective disulfide bond reducing agent [10, 11], before incubation with *H*. *somni* (Fig 3B). DTT did not show any adverse effect on bacterial growth, but rather enhanced bacterial growth as compared to bacteria incubated only with PBS (negative control).

**Fig 4:**
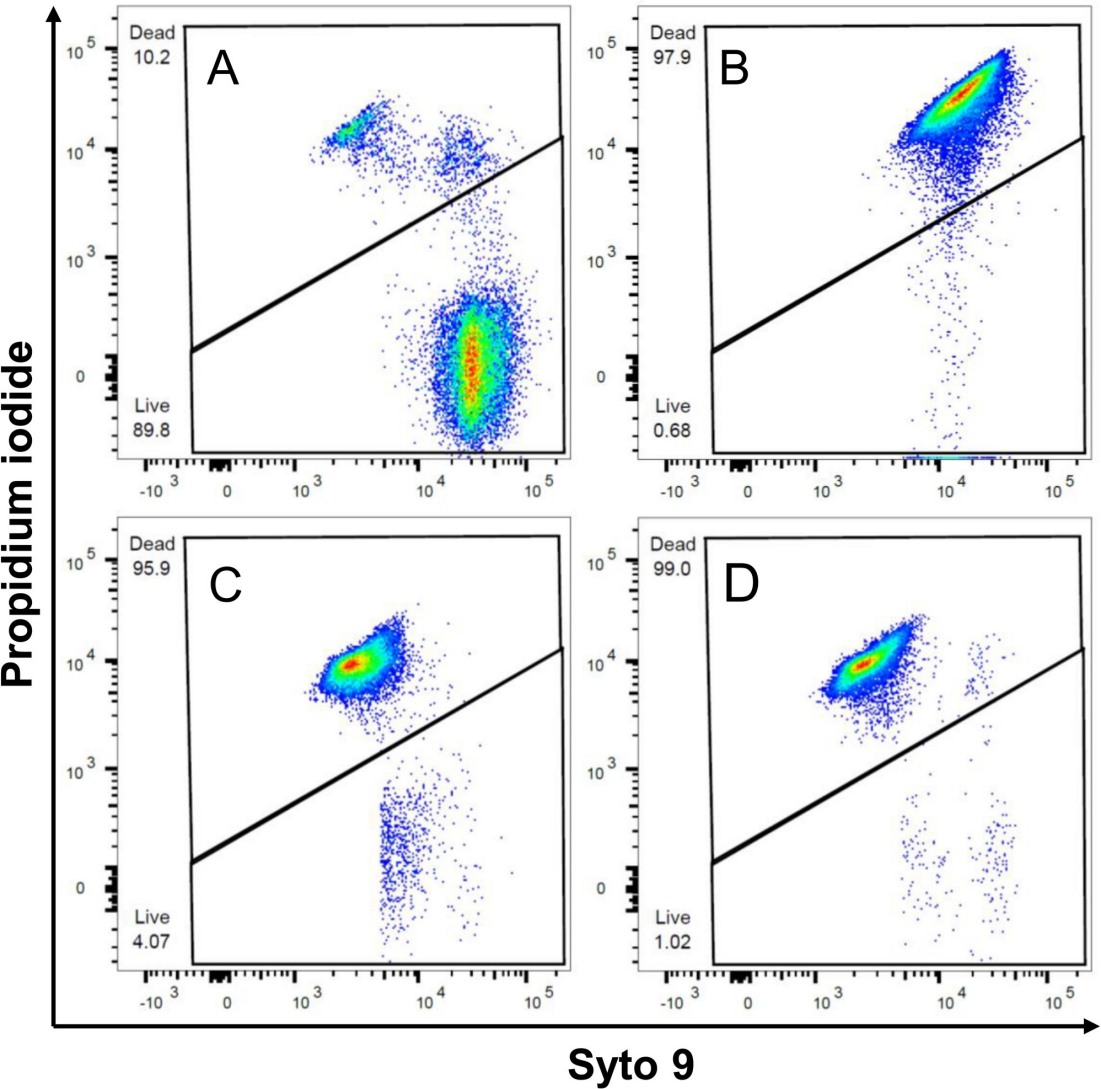
Flow cytometric analysis confirmed that both bNK2A peptides induced damage to *Histophilus somni* membranes. Live and dead *H*. *somni* in PBS control (A, PBS), heat-killed at 56°C for 10 min (B), and treated with 20 μM bNK2A-C10C20 (C), or 20 μM bNK2A-S10S20 (D) at 37°C for 30 min were stained with LIVE/DEAD *Bac*Light bacterial viability dyes. X-axis indicates live bacteria (Syto 9) and Y-axis indicates dead bacteria (propidium iodide). Results of one representative experiment out of three are shown.

**Fig 5:**
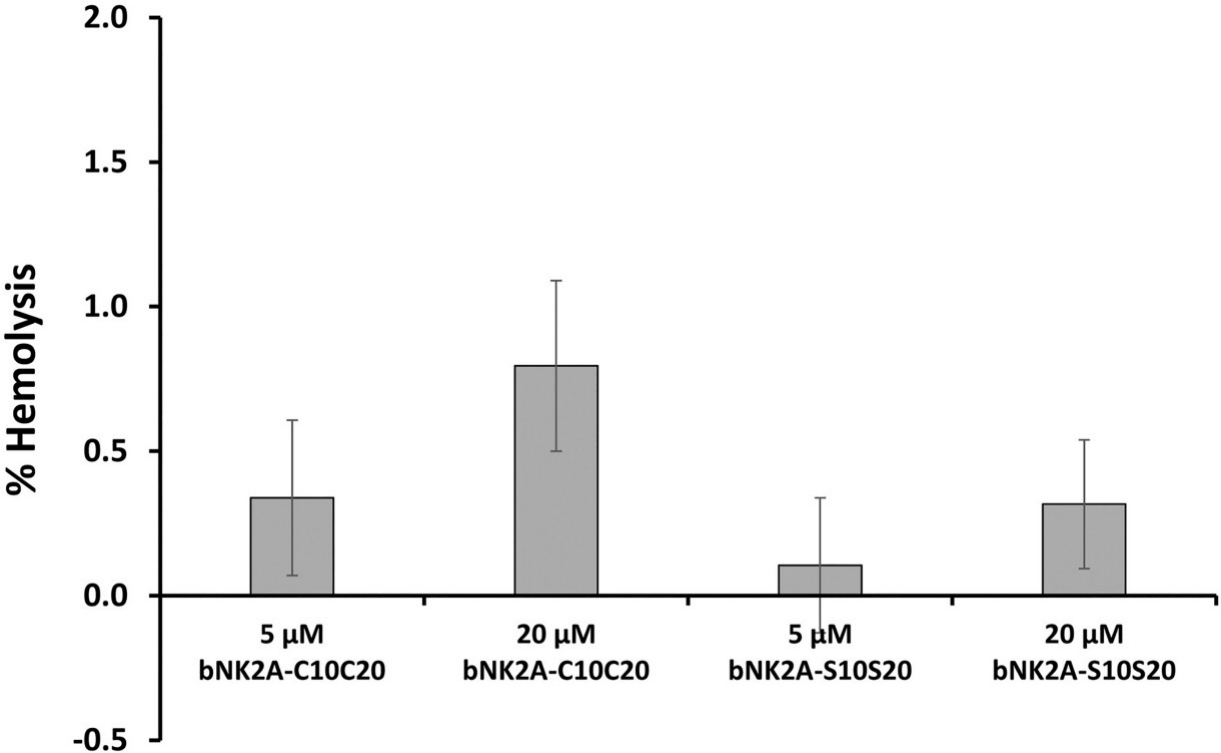
Both bNK2A peptides showed minimal hemolytic activity against cattle erythrocytes. The cattle RBCs resuspended in PBS (2.5% hematocrit) were incubated with original bNK2A-C10C20 or analog bNK2A-S10S20 peptides (5 μM and 20 μM final concentrations) at 37°C in a humidified atmosphere for 60 min. The released hemoglobin was spectrophotometrically measured at 405 nm wavelength using a microplate reader. RBCs incubated with 0.1% (v/v) Triton X-100 or PBS served as positive and negative controls, respectively. Means and SD were calculated from two independent experiments.

Two color flow cytometry analysis was performed on *H*. *somni* incubated with bNK2A peptides after staining with LIVE/DEAD *Bac*Light bacterial viability dyes [15]. Results of flow cytometric analysis of *H*. *somni* incubated with both peptides were consistent with killing assay findings (Fig 4 and Table 1). The strong PI staining of *H*. *somni* with the original as well as the analog bNK2A-S10S20 peptide confirms both peptides were equally effective in killing by bacterial membrane pore formation. Indeed, the pore formation properties of bNK2A-C10C20 on *H*. *somni* inner- and outer-membranes have been previously confirmed by us using electron microscopy [14]. The lack of hemolytic activity of the original bNK2A-C10C20 peptide has also previously been reported by us [15]. Both bNK2A peptides showed <2% hemolysis at 5 μM and 20 μM final concentrations against cattle erythrocytes (Fig 5). Therefore, due to the lower hemolytic activity of bNK2A peptides on erythrocytes and strong antimicrobial activity against *H*. *somni*, bNK2A peptides appear to have selective activity against bacterial membranes.

**Table 1:** Mean percentages of dead *Histophilus somni* in bNK2A peptides treated samples as assessed by flow cytometry.

	Control	Heat killed	bNK2A-S10S20	bNK2A-C10C20
Mean*	8.26	98.3	96.57	96.89
SD	3.62	1.54	1.83	3.89

*H. *somni* was incubated with 20 μM bNK2A peptides for 30 min followed by staining with LIVE/DEAD *Bac*Light bacterial viability dyes before flow cytometry analysis.

The loss of antimicrobial activity was reported for NK-lysin peptides from pigs and *E*. *histolytica* upon pre-incubation with reducing agents [11, 12]. However, similar to our findings, pre-incubation with a reducing agent (DTT) or replacement of cysteines with serines of synthetic human granulysin-derived peptides also failed to reduce bactericidal activity [10]. Similarly, reduction of disulfide bonds of guinea pig cation peptides with DTT also failed to reduce its bacterial killing activity [19]. A recent study also found that disulfide bond in a thanatin peptide is not essential for its antimicrobial activity [13]. The mature region of granulysin and NK-lysins have five α-helices with six well-conserved cysteines which are expected to form intra-chain disulfide bonds. However, reduction of disulfide bonds in recombinantly expressed mature granulysin protein (~9kDa) or replacement of cysteines in mature granulysin protein with serines failed to reduce but rather increased lytic activities [10]. Similar studies are yet to be performed with recombinantly expressed mature bovine NK2A protein. Therefore, findings in this study suggest that two cysteines or disulfide bonds appeared not to be essential for potent antimicrobial activity of synthetic 30-mer bNK2A peptide.

## Conclusions

In order to understand the role of disulfide bonds of bNK2A peptide on antimicrobial activity, we used several techniques such as CD spectroscopy, mass spectrometry, bacterial killing assay, and flow cytometry. Findings from these assays suggested that neither two cysteines nor intra-chain disulfide bond formation between helices 2 and 3 of the synthetic bNK2A-C10C20 peptide is crucial for potent bNK2A bactericidal activity. Due to the lack of widespread bacterial resistance mechanism(s) to AMPs, AMPs have been recognized as a potential novel therapeutic agent (or alternative to antibiotics) for treating emerging multidrug resistant bacterial infections. Several AMPs such as hLF1-11, LL-37, omiganan, pexiganan, and PAC-113 are currently in the phase I/II/III clinical trials [20]. Further modifications to the bNK2A peptides and *in vitro* bacterial killing assays are currently underway in our laboratory to develop a clinically suitable bNK2A peptide to evaluate its antibacterial activity in animal models of BRDC.
